# Anlotinib suppresses tumor progression via blocking the VEGFR2/PI3K/AKT cascade in intrahepatic cholangiocarcinoma

**DOI:** 10.1038/s41419-020-02749-7

**Published:** 2020-07-24

**Authors:** Fei Song, Bo Hu, Jian-Wen Cheng, Yun-Fan Sun, Kai-Qian Zhou, Peng-Xiang Wang, Wei Guo, Jian Zhou, Jia Fan, Zhong Chen, Xin-Rong Yang

**Affiliations:** 1 Department of Hepatobiliary Surgery, Affiliated Hospital of Nantong University, Nantong, 226001 PR China; 2 Department of Liver Surgery and Transplantation, Liver Cancer Institute, Zhongshan Hospital, Fudan University; Key Laboratory of Carcinogenesis and Cancer Invasion, Ministry of Education, Shanghai, 200032 PR China; 3 Department of Laboratory Medicine, Zhongshan Hospital, Fudan University, Shanghai, 200032 PR China; 4 Institutes of Biomedical Sciences, Fudan University, Shanghai, 200032 PR China

**Keywords:** Cancer, Molecular biology

## Abstract

Intrahepatic cholangiocarcinoma (ICC) is a malignant tumor derived from bile duct epithelium. Its characteristics include an insidious onset and frequent recurrence or metastasis after surgery. Current chemotherapies and molecular target therapies provide only modest survival benefits to patients with ICC. Anlotinib is a novel multi-target tyrosine kinase inhibitor that has good antitumor effects in a variety of solid tumors. However, there are few studies of anlotinib-associated mechanisms and use as a treatment in ICC. In this study using in vitro experiments, we found that anlotinib had significant effects on proliferation inhibition, migration and invasion restraint, and cell-cycle arrestment. Anlotinib treatment affected induction of apoptosis and the mesenchymal–epithelial transition. Patient-derived xenograft models generated directly from patients with ICC revealed that anlotinib treatment dramatically hindered in vivo tumor growth. We also examined anlotinib’s mechanism of action using transcriptional profiling. We found that anlotinib treatment might mainly inhibit tumor cell proliferation and invasion and promote apoptosis via cell-cycle arrestment by inactivating the VEGF/PI3K/AKT signaling pathway, as evidenced by significantly decreased phosphorylation levels of these kinases. The activation of vascular endothelial growth factor receptor 2 (VEGFR2) can subsequently activate PI3K/AKT signaling. We identified VEGRF2 as the main target of anlotinib. High VEGFR2 expression might serve as a promising indicator when used to predict a favorable therapeutic response. Taken together, these results indicated that anlotinib had excellent antitumor activity in ICC, mainly via inhibiting the phosphorylation level of VEGFR2 and subsequent inactivation of PIK3/AKT signaling. This work provides evidence and a rationale for using anlotinib to treat patients with ICC in the future.

## Introduction

Intrahepatic cholangiocarcinoma (ICC) is the second most common primary hepatic malignancy after hepatocellular carcinoma. ICC incidence and mortality rates have been rapidly increasing worldwide^1–3^. Surgical resection remains the mainstay of potentially curative therapy for ICC patients. However, the high rates of recurrence or metastasis (about 70% after surgery) after resection result in dismal outcomes for patients with ICC (5-year survival rate <30%)^4–6^. Most patients present with unresectable or distant metastatic disease at the time of diagnosis, and no effective chemotherapies or molecular target therapies are available for these patients^7–9^. The standard systemic therapy for ICC is platinum-based chemotherapy combined with gemcitabine, but it provides only a modest survival benefit^10^. Thus, it is urgent to find new and effective treatments for patients with ICC.

Anlotinib is a multi-targeted receptor tyrosine kinase inhibitor (TKI) with antitumor effects in a variety of solid tumors^11^. Anlotinib inhibits tumor growth by inhibiting signaling pathways involved in angiogenesis and cell proliferation^12,13^. Phase-II and -III clinical trials found that anlotinib has beneficial clinical activity for patients with non-small cell lung cancer, hepatocellular carcinoma, renal carcinoma, gastric cancer, and soft tissue sarcoma^11,14,15^. Preclinical and clinical trials have also found that several similar antiangiogenic TKIs (e.g., regorafenib, lenvatinib, and apatinib) have promising antitumor activity in animals models and in patients with cholangiocarcinoma^16–18^. Given the advanced disease for most unresectable ICC cases, anlotinib might be a new opportunity for those patients and have a better therapeutic effect by prolonging progression-free survival times and overall survival times.

The efficacy of anlotinib and its potential molecular mechanisms remain to be fully characterized. This study is the first to examine the therapeutic anlotinib effects on ICC and its possible molecular mechanisms. This work provides promising evidence and rationale for clinical therapeutic application of anlotinib in human patients with ICC.

## Results

### Anlotinib inhibits ICC cell growth and promotes apoptosis in vitro

**Fig. 1:**
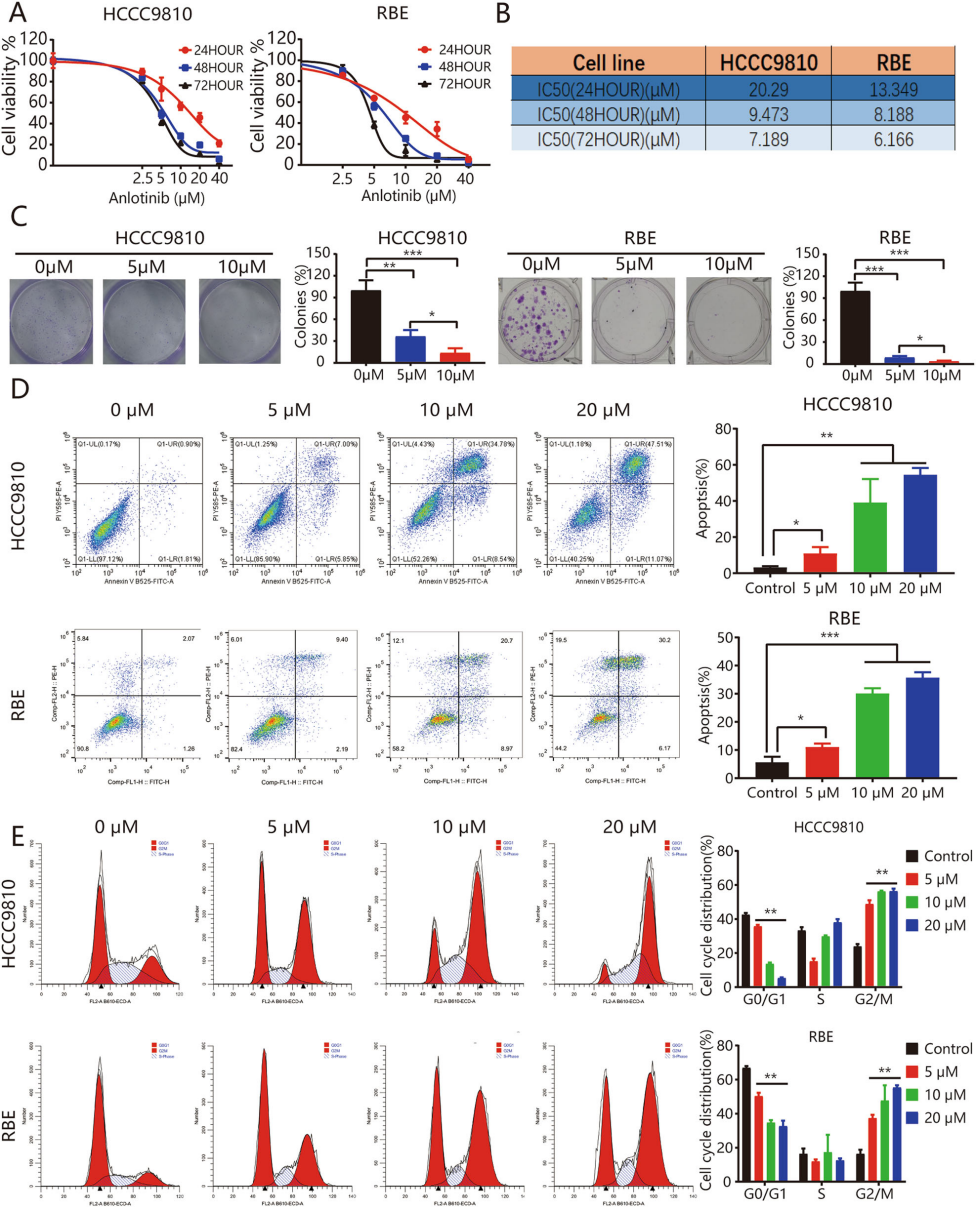
Anlotinib inhibits ICC cell growth and invasion, promotes apoptosis in vitro. **a**, **b** HCCC9810 and RBE cells were treated with anlotinib at the indicated concentrations for 24, 48, or 72 h. Cell viability was measured using CCK8, and IC50 values were calculated; **c** representative images of the clone formation assay; **d** the ratio of apoptotic cells was measured in RBE and HCCC9810 cells after anlotinib treatment for 48 h. Apoptosis was detected using Annexin V-FITC and propidium iodide (PI) staining; **e** cells were treated with anlotinib at the indicated concentration for 24 h and then stained with PI for flow cytometric analysis. Each experiment was performed in triplicate. *P < 0.05; **P < 0.01.

To evaluate anlotinib effects, we incubated human ICC cell lines (HCCC9810 and RBE) with or without anlotinib at specific concentrations (5, 10, or 20 μM) for 24, 48, and 72 h. We used the CCK8 assay to assess tumor cell viability and found that anlotinib inhibited tumor cell growth significantly in a dose- and time-dependent manner (Fig. 1a). The results for the IC50 values for the two cell lines are presented in Fig. 1b. Colony formation assays revealed that anlotinib significantly inhibited clone numbers in the HCCC9810 and RBE cell lines (all *P* < 0.05; Fig. 1c). We used flow cytometry (FCM) to examine anlotinib effects on tumor cell apoptosis. In both cell lines, the apoptosis rates increased significantly after anlotinib administration (all *P* < 0.05; Fig. 1d). Cell cycle analysis revealed that anlotinib administration in HCCC9810 cells induced G2/M phase arrest, accompanied by decreases in the numbers of cells in the G1 phase (Fig. 1e). Similar results occurred in the RBE cell line (Fig. 1e).

### Anlotinib inhibits migration and invasion of ICC cells via inhibition of the epithelial–mesenchymal transition (EMT)

**Fig. 2:**
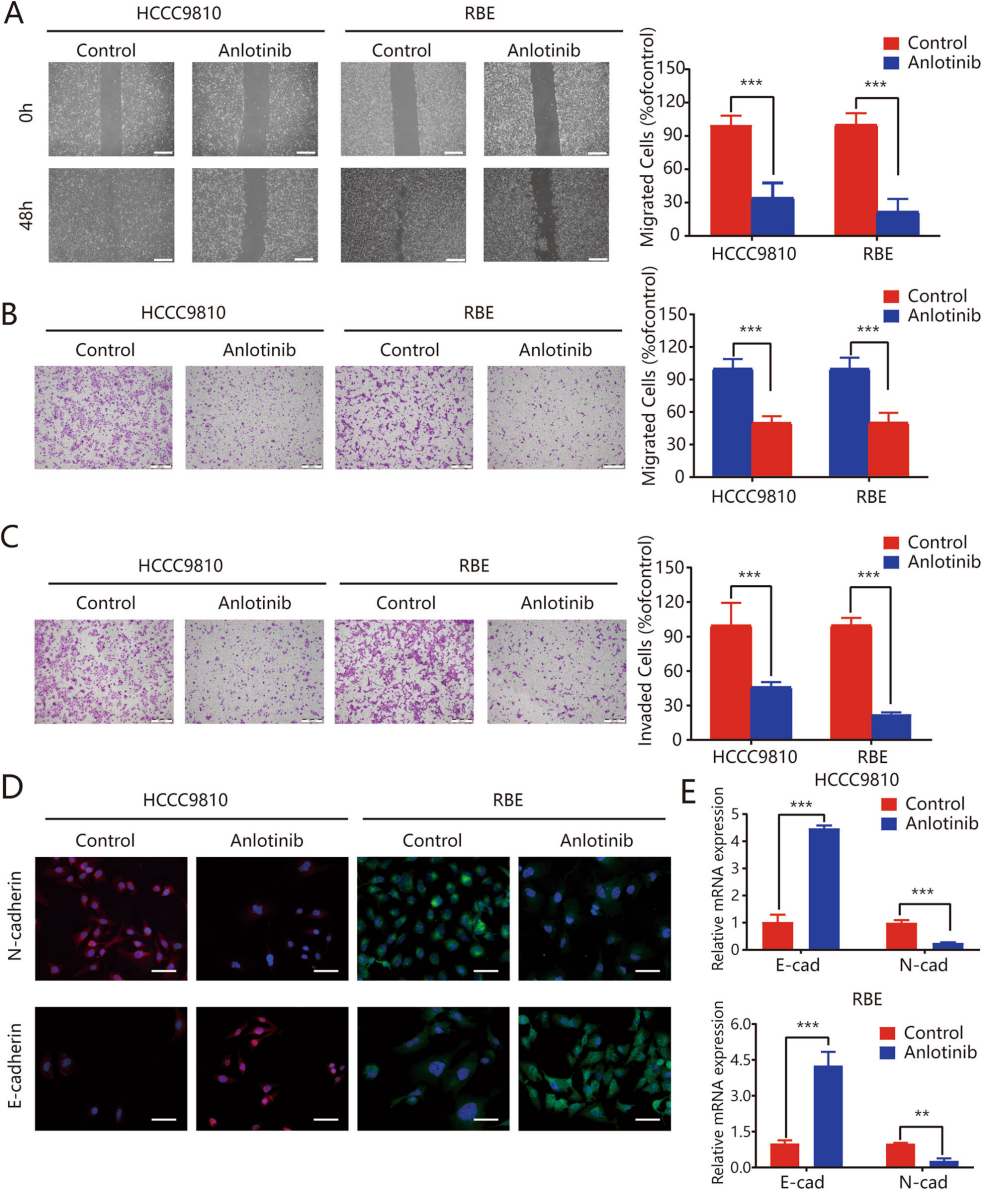
Anlotinib inhibits migration and invasion of ICC cells via inhibition of the epithelial–mesenchymal transition. The effects of anlotinib on cell invasion and migration were measured using wound-healing (**a**) and transwell assays without Matrigel (**b**) and with Matrigel (**c**); **d** after treatment with anlotinib, expression and localization of E-cadherin and N-cadherin markers in HCCC9810 and RBE cells were analyzed using immunofluorescence staining; **e** the expression of EMT markers (E-cadherin and N-cadherin) in ICC cell lines were detected using qRT-PCR analyses. *P < 0.05; **P < 0.01; ***P < 0.001. Scale bars = 100 μm.

We evaluated the inhibitory effects of anlotinib on ICC cells using wound-healing and transwell assays. The wound-healing assays revealed that anlotinib effectively inhibited the migration of HCCC9810 cells (100.00 ± 4.14 for control vs. 34.32 ± 6.71 for anlotinib, *P* < 0.01); similar results were observed for the RBE cell line (100.00 ± 5.21 vs. 21.84 ± 5.73, respectively, *P* < 0.01; Fig. 2a). The transwell assay results indicated that anlotinib administration markedly decreased the numbers of migrating and invading HCCC9810 and RBE tumor cells (all *P* < 0.01; Fig. 2b, c).

We used uantitative real-time polymerase chain reaction (qRT-PCR), westen blot, and immunofluorescence (IF) assays to assess the levels of EMT marker expression. Downregulation of N-cadherin, Vimentin, αSMA, CK19, and upregulation of E-cadherin (epithelial marker) occurred after anlotinib treatment of the HCCC9810 and RBE cell lines (Figs. 2d, e and S1). Collectively, these results indicated that anlotinib inhibited migration and invasion of ICC cells via EMT inhibition.

### Anlotinib exhibits effective antitumor activity with low drug toxicity in patient-derived tumor xenograft (PDX) models

**Fig. 3:**
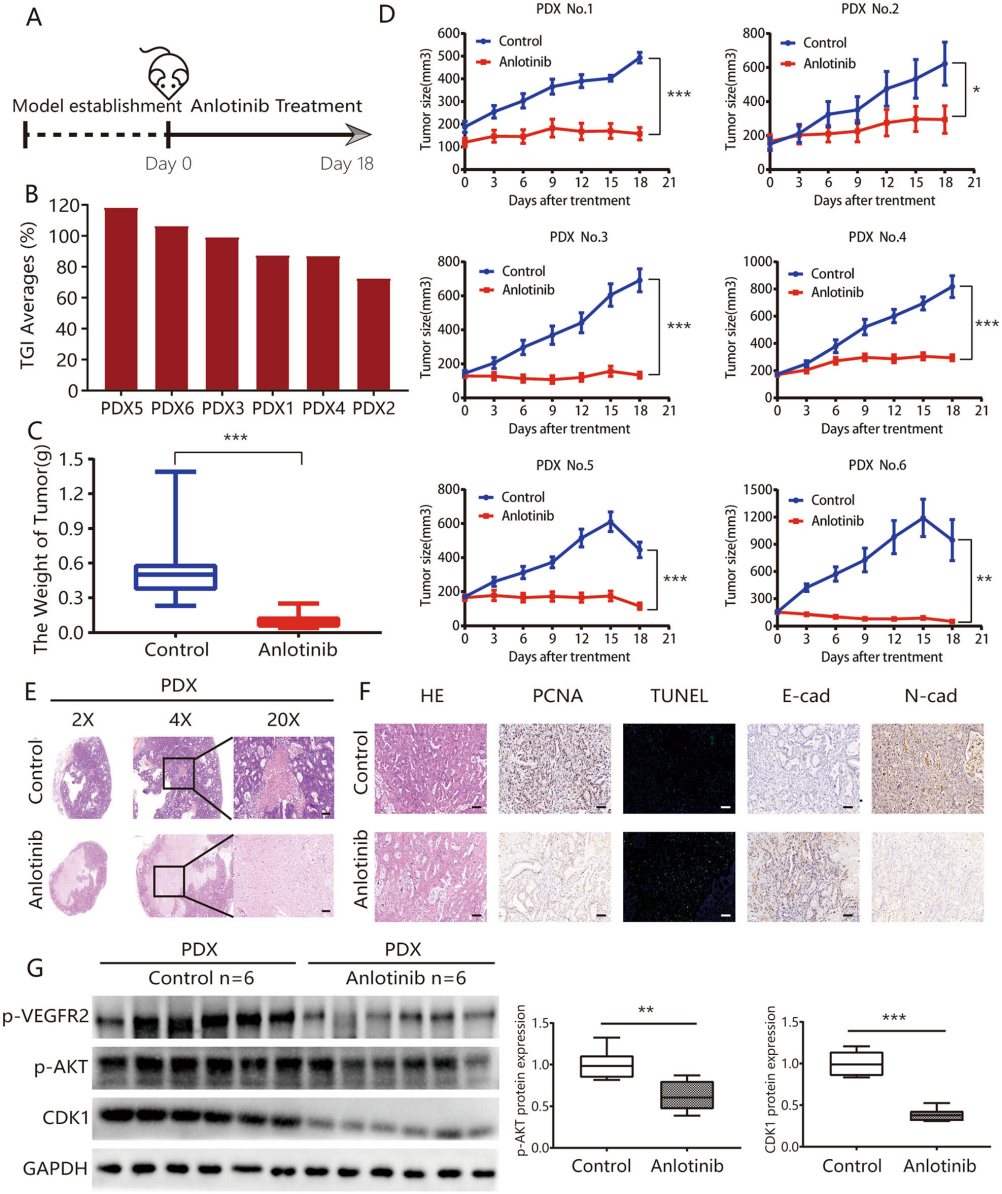
Anlotinib exhibits effective antitumor activity with low drug toxicity in PDX models. **a** Timeline of the experiments in vivo; **b** waterfall plot of anlotinib response after treatment in six PDX models; **c** there were significant differences in tumor weights between the anlotinib treatment group and the control group (0.58 ± 0.08 vs. 0.10 ± 0.01, respectively); **d** tumor growth curves for each PDX case; **e** representative hematoxylin and eosin staining of tumor tissues from PDX models, with or without anlotinib treatment; **f** anlotinib treatment group had substantial decreases in proliferation (PCNA) rates and increases in apoptosis (TUNEL) index numbers, compared with the control group. The expression of E-cadherin was significantly downregulated, while the expression of N-cadherin was upregulated, based on the immunohistochemistry (IHC) assay after anlotinib treatment; **g** expression of p-VEGFR2, p-AKT, CDK1 in PDX tumors was detected using western blot assay. *P < 0.05; **P < 0.01; ***P < 0.001. Scale bars = 100 μm.

We further explored anlotinib effects in vivo based on six previously constructed ICC PDX models (Fig. 3a). The results for tumor inhibition rate (TGI) indicated that the average TGI values in six PDX models were 118.20, 106.30, 99.20, 87.40, 87.10, and 73.00% (average of the six models, 95.20%; Figs. 3b and S2A). Tumor weights in the control group were significantly greater than those in the anlotinib treatment group (0.58 ± 0.08 vs. 0.10 ± 0.01, respectively, *P* < 0.001; Fig. 3c). The growth curves of representative PDX models treated using phosphate-buffered saline (PBS; control) or anlotinib indicated that the mice in the control group had rapid and significant tumor growth, compared with the anlotinib-treated mice (all *P* < 0.05; Fig. 3d). Histopathological examination (H&E) of the tumor tissues revealed that the tumors in the anlotinib-treated group had more necrotic foci than those in the control group (Fig. 3e). We found that anlotinib administration for 18 days had no significant effect on body weight in the PDX mouse model (Fig. S2B). There were also no significant changes with anlotinib administration in diarrhea, appetite, or mental state in the PDX mouse model. Taken together, these results indicated that anlotinib had effective antitumor activity and low drug toxicity in vivo.

Biomarkers for tumor cell proliferation, apoptosis, and the EMT were also detected using the PDX models (Figs. 3f and S3). The anlotinib treatment group had a significant decrease in proliferation rate and an increase in the apoptosis index. The immunohistochemistry (IHC) assay revealed that anlotinib treatment resulted in significant downregulation of N-cadherin expression and upregulation of E-cadherin expression. These results were consistent with the results of the in vitro experiments.

### Transcriptome analysis indicates that anlotinib is involved in the VEGF/PI3K/AKT signaling pathway in ICC cells

**Fig. 4:**
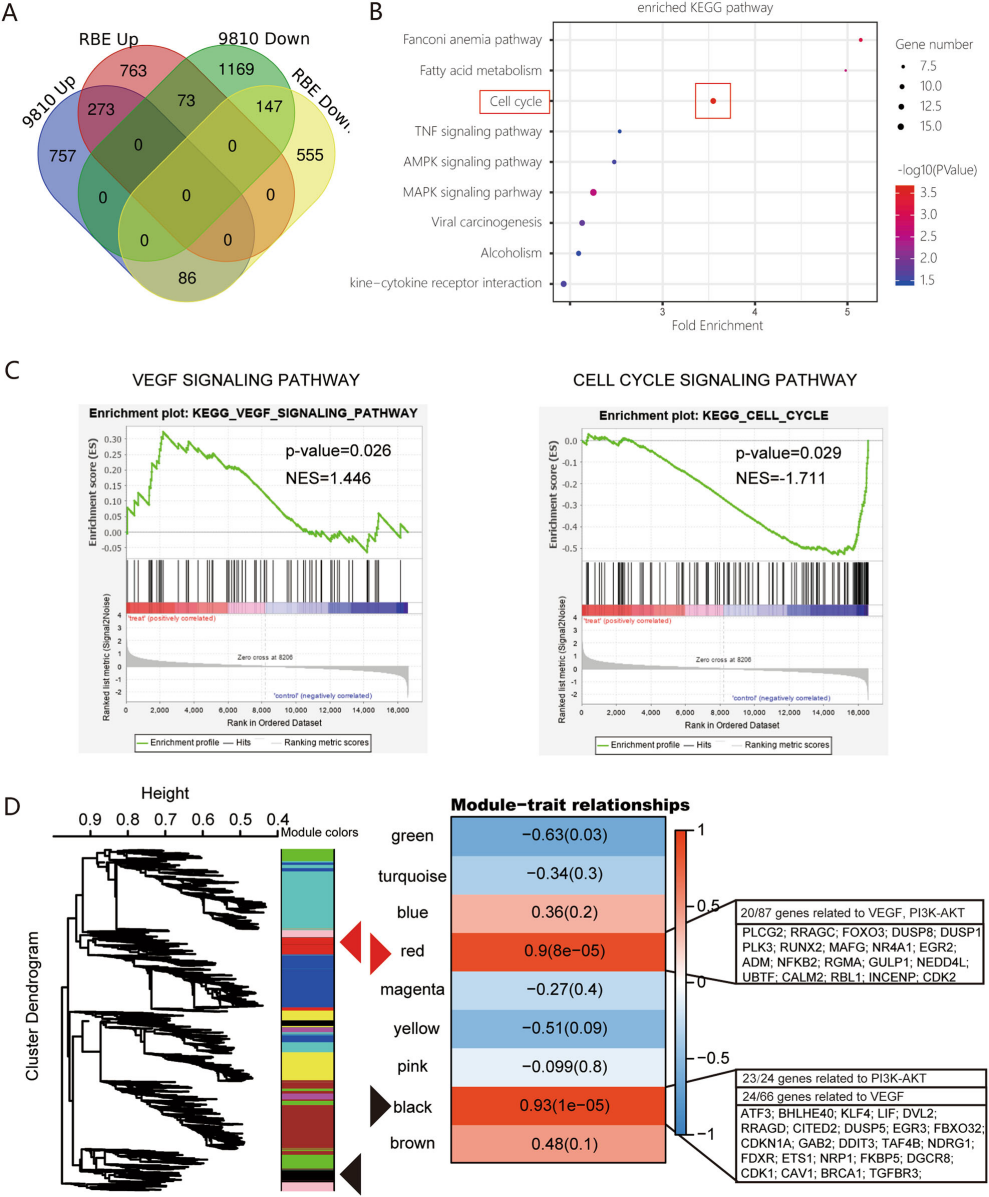
Transcriptome analysis indicates the effect of anlotinib in ICC is via the VEGF/PI3K/AKT signaling pathway. **a** Venn diagram revealed the common up/downregulated genes (adjusted *P* value < 0.05, fold-change = 2) detected using RNA-seq in HCCC9810 and RBE cells treated with anlotinib; 420 genes were shared in the two cell lines (273 genes upregulated, 147 genes downregulated); **b** KEGG enrichment analysis based on these differentially expressed genes; **c** gene set enrichment analysis of mRNA expression profiles of the two cell lines revealed that cell cycle and VEGF signaling pathways were significantly altered after anlotinib treatment of ICC cells; **d** a weighted gene co-expression network analysis algorithm was implemented to construct the gene co-expression network. The module–trait relationships revealed two modules highly correlated with anlotinib treatment phenotype: the black module (correlation: 0.93, *P* < 0.01) and the red module (correlation: 0.90, *P* < 0.01). Pathway annotation showed that the genes in these two critical modules were mainly enriched in the VEGF signaling network and PI3K/AKT signaling pathway.

To examine potential mechanisms of anlotinib effects, we used RNA-seq to analyze anlotinib treatment effects on transcriptional alterations in ICC cell lines. HCCC9810 and RBE cells were treated with DMSO or anlotinib at 5 μM for 24 h, followed by total RNA isolation and deep sequencing. We used a Venn diagram of the results of RNA-seq of HCCC9810 and RBE cells treated with anlotinib to indicate the genes that were upregulated or downregulated (adjusted *P* value < 0.05, fold-change = 2.0) (Fig. 4a). The two groups shared 420 genes (Fig. 4a, 273 genes upregulated, 147 genes downregulated). The KEGG enrichment analysis based on these altered genes revealed that the cell cycle signal pathway was the most significantly enriched pathway (Fig. 4b). Gene set enrichment analysis of the mRNA expression profile of the two cell lines also revealed that cell cycle signature genes were negatively enriched after anlotinib treatment (Fig. 4c). The putative anlotinib target pathway, vascular endothelial growth factor (VEGF) signaling pathway^11,12^, was also significantly altered after anlotinib treatment of two ICC cell lines (Fig. 4c).

To further identify co-expression modules related to anlotinib treatment, we used a weighted gene co-expression network analysis algorithm to construct a gene co-expression network^19,20^. The module–trait relationships revealed that two modules were highly correlated with anlotinib treatment phenotype, the black module (correlation: 0.93, *P* < 0.01; Fig. 4d) and the red module (correlation: 0.90, *P* < 0.01; Fig. 4d). Pathway annotation revealed that the genes in these two critical modules were enriched in the VEGF signaling network and the PI3K/AKT signaling pathway, which have key roles in control of cell cycle progression^21^ (24 of 66 in black module, and 20 of 87 in the red module). Taken together, the results of previous studies about these two key signal pathways in tumors^22,23^ combined with our results suggested that anlotinib is involved in the VEGF/PI3K/AKT signaling pathway inactivation.

**Fig. 5:**
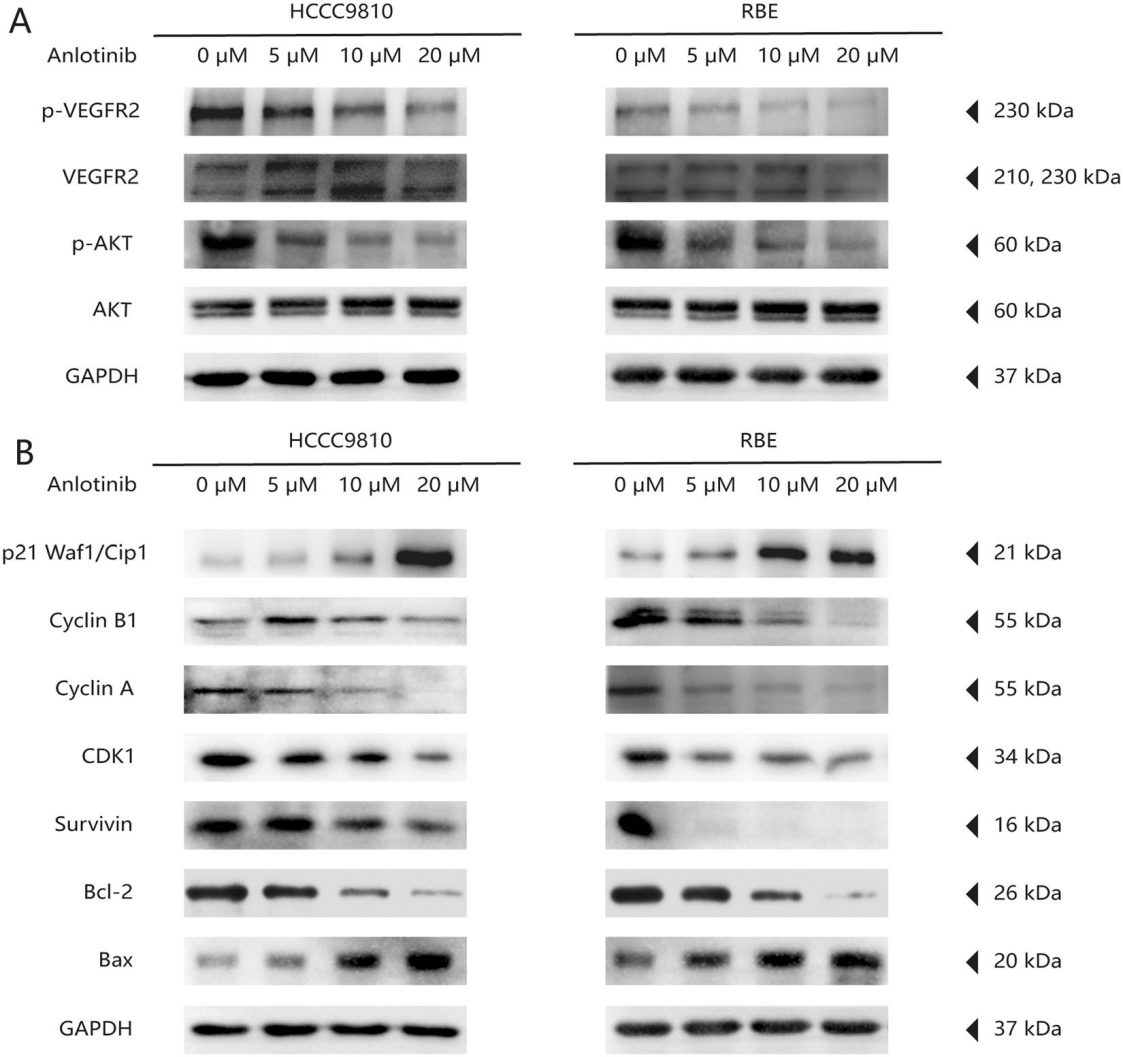
Anlotinib affects the VEGF/PI3k/AKT signaling pathway. **a** The expression and phosphorylation levels of VEGFR2 and AKT in ICC cell lines after treatment with concentration gradient anlotinib were detected using the western blot assay; **b** expression of cell-cycle-related and apoptosis-related proteins in ICC cell lines after treatment with concentration gradient anlotinib for 24 h was detected using immunoblotting.

We further evaluated the protein levels and phosphorylation of three common anlotinib targets (i.e., VEGFR2, fibroblast growth factor receptor 1 (FGFR1), and platelet-derived growth factor receptor-β (PDGFR-β))^24^. We found that the phosphorylation levels of VEGFR2 and AKT were significantly reduced in the anlotinib group compared with the control group (all *P* < 0.05; Figs. 5a and S4A). The between-group difference in VEGFR2 protein levels was not significant (*P* > 0.05; Figs. 5a and S4A). However, we found that FGFR1 and PDGFR-β protein levels and their phosphorylation remained unchanged between the two groups (all *P* > 0.05; Fig. S5A). We further examined expression of cell-cycle-related proteins and found that in the anlotinib group, expression of cyclin-dependent kinases (CDK1), cyclin A, and cyclin B1 were decreased significantly, while expression of p21 Waf1/Cip1 was significant upregulated^25^ (all *P* < 0.05; Figs. 5b and S4B). We also found that the protein level of Bax was significantly upregulated, and protein levels of Bcl-2 and Survivin^26^ were significantly decreased (all *P* < 0.05; Figs. 5b and S4B). Taken together, these results suggested that anlotinib treatment inhibited tumor cell proliferation and invasion and promoted apoptosis via cell cycle arrestment by inactivating the VEGF/PI3K/AKT signaling pathway.

### Anlotinib restrains the PI3K/AKT signaling pathway via inhibition of the level of VEGFR2 phosphorylation in ICC cells

**Fig. 6:**
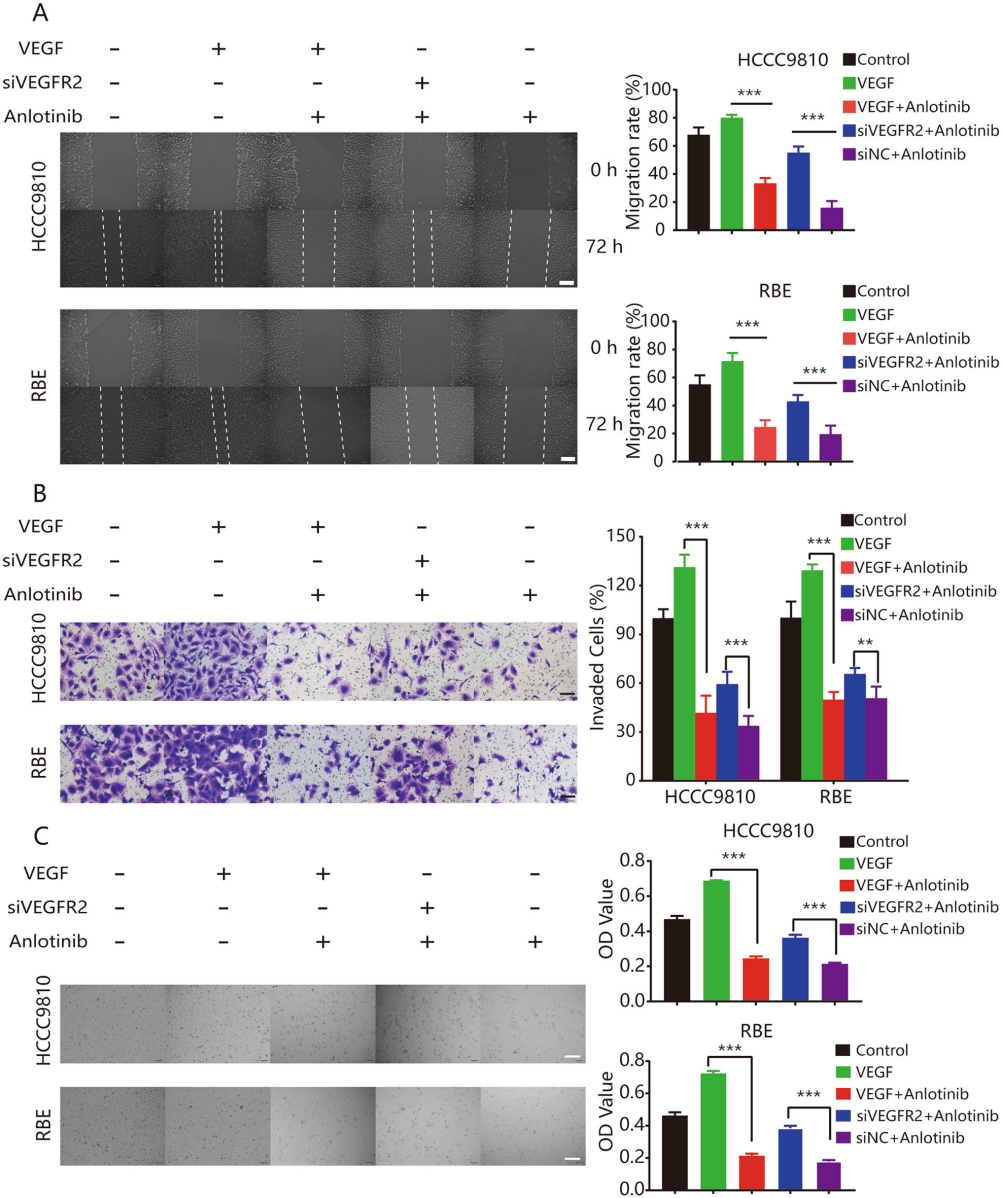
Antitumor activity of Anlotinib was mainly dependent on VEGFR2 expression. **a** Wound-healing assays showed the effects of VEGFR2 expression on the anti-migration ability of anlotinib. Representative images of migration are shown in the left panel. Degrees to which wounds healed in the indicated groups are shown in the histogram. Significantly decreased inhibition effects on migration of anlotinib were observed when VEGFR2 was silenced in HCCC9810 and REB ICC cells lines; **b** transwell assays revealed the effects of VEGFR2 expression on the anti-invasion ability of anlotinib. Inhibition effects of anlotinib were significantly reduced when VEGFR2 expression was knocked down. The histogram shows the relative proportions of invading cells; **c** CCK8 experiments were performed to confirm the effects of VEGFR2 in anlotinib-induced inhibition of proliferation. When VEGFR2 expression was knocked down, anlotinib’s antiproliferative ability was significantly reduced. Representative images were photographed using an inverted microscope and are shown in the left panel. The histogram shows the OD values for each group after 48 h of drug treatment. *P < 0.05; **P < 0.01; ***P < 0.001. Scale bars = 100 μm.

**Fig. 7:**
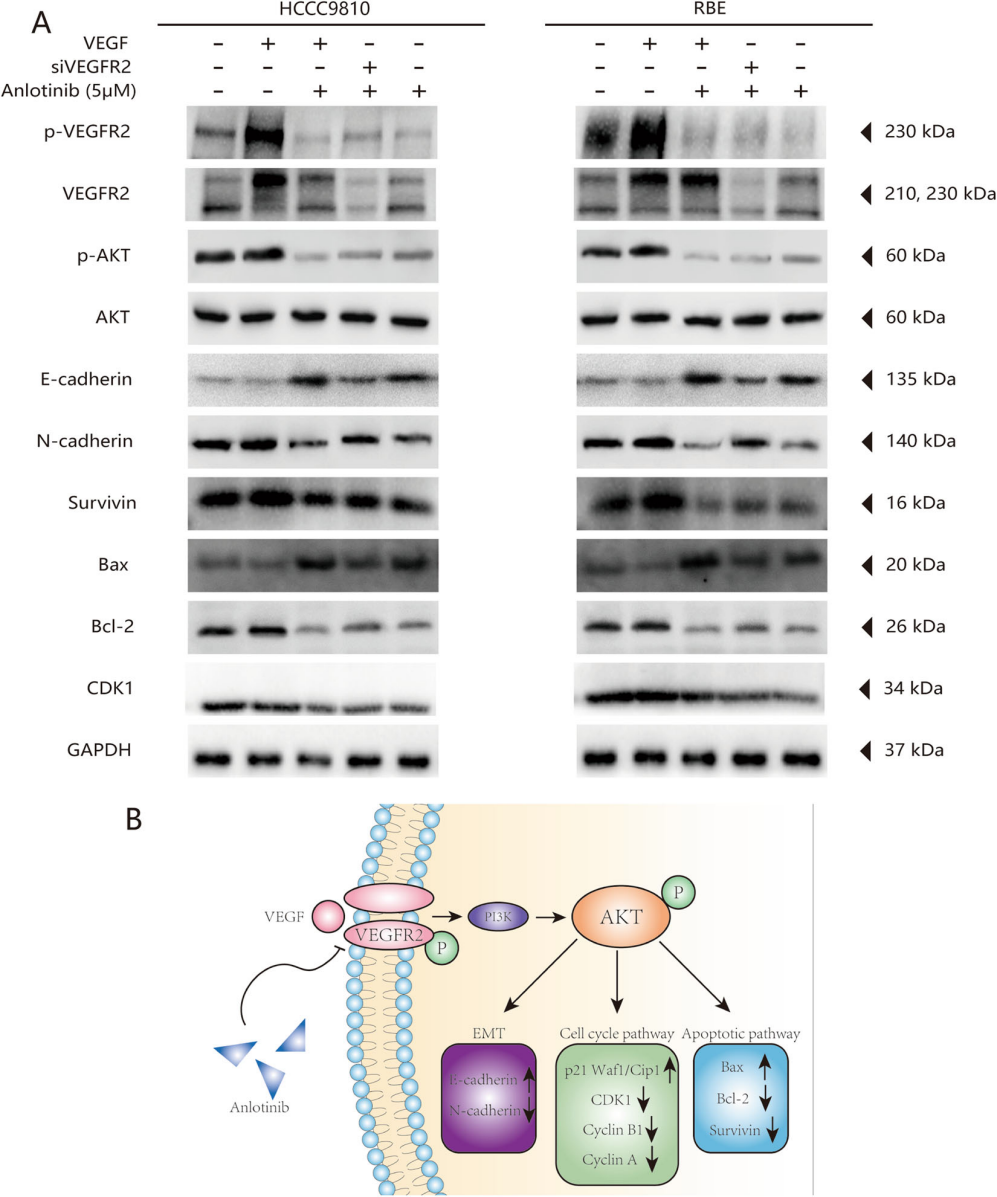
Anlotinib restrains the PI3K/AKT signaling pathway via inhibition of VEGFR2 phosphorylation levels in ICC cells. **a** Western blot assay was used to detect the expression of cell-cycle-related and apoptosis-related proteins and the phosphate expression of VEGFR2 and AKT in ICC cells in different treatment groups; **b** schematic depiction of the underlying mechanism of anlotinib-inhibiting tumor activity in ICC cells via the VEGFR2/PI3K/AKT signaling pathway.

Based on the results of the transcriptome-wide differential gene expression analysis, we further evaluated the role of VEGFR2 in anlotinib-induced antitumor effects via VEGFR2 silencing. We used a small interfering RNA duplex (siVEGFR2) in the HCCC9810 and RBE cell lines. We found that anlotinib stopped the proliferation, migration, and invasion effects in the ICC cells that were caused by recombinant human VEGF (rhVEGF); siVEGFR2 decreased the effects of anlotinib in the ICC cells (Fig. 6a–c). To further investigate the potential molecular mechanisms of anlotinib, we performed western blot analysis to detect expression levels of key proteins in the VEGFR2/PI3K/AKT signaling pathway. We compared the control, VEGF-treated, anlotinib plus VEGF-treated, siVEGFR2 plus anlotinib, and the negative control small interfering RNA (siNC) plus anlotinib groups. As shown in Fig. 7a, we found that anlotinib abolished VEGF-induced phosphorylation of VEGFR2 and AKT in the two ICC cell lines. This change caused subsequent corresponding changes in key proteins in downstream genes, including E-cadherin, N-cadherin, Bax, Bcl-2, Survivin, and CDK1 (Fig. 7a). Compared with the VEGF-treated group, expression of CDK1, Survivin, Bcl-2, and N-cadherin was decreased significantly, while Bax and E-cadherin expression was significantly upregulated in the anlotinib plus VEGF-treated group (Fig. 7a). However, we also found that compared with the siNC plus anlotinib group, the protein levels of CDK1, Survivin, Bcl-2, and N-cadherin were also upregulated, while Bax and E-cadherin expression were decreased in the siVEGFR2 plus anlotinib group (Fig. 7a). Taken together, these results suggested that anlotinib inhibited the proliferation, migration, and invasion of ICC cells, primarily through the VEGFR2/PI3K/AKT signaling pathway.

The results of the PDX tumor tissue model indicated that in the anlotinib group the phosphorylation levels of VEGFR2 and AKT and the protein levels of the CDK1 group were also significantly decreased, compared with the control group (Fig. 3g). We also examined expression of VEGFR2 before anlotinib treatment and found that VEGFR2 expression before anlotinib treatment showed a significant positive correlation with the TGI with anlotinib treatment (*r* = 0.671, *P* = 0.046; Fig. S5B, S5C).

Collectively, our results indicated that the therapeutic anlotinib effects occurred mainly through inhibiting VEGFR2 phosphorylation levels and then affecting AKT phosphorylation. These actions then caused the changes in downstream genes associated with tumor cell proliferation, invasion, and apoptosis (Fig. 7b).

## Discussion

ICC accounts for 10–15% of all cases of primary liver cancer, with an increasing trend in incidence in recent years^27^. There is no effective drug treatment strategy for patients with ICC. Anlotinib is a new, small, orally administered multi-target TKI that was independently developed in China. Its excellent antitumor effects have been confirmed for several types of cancer^28–30^. In this study, we found that anlotinib had excellent antitumor effects in ICC cell lines and ICC PDX models. We also found that the antitumor effect of anlotinib was likely via inhibition of the phosphorylation level of VEGFR2 and subsequent activation of the PI3K/AKT signaling pathway.

In this study, the antitumor activity of anlotinib in vivo was supported by the results for two ICC cell lines. The IC50 of anlotinib was much lower than that for other reported TKI agents in ICC cell lines^31^. This result suggested that anlotinib may have a better response. The results using six ICC PDX models also supported anlotinib’s excellent antitumor effects (Fig. 3b, c). These models are valid preclinical models for oncology drug development and drug response prediction^32^. After administration of anlotinib in six PDX models at a dosage of 3 mg/kg/day, we found that treatment with anlotinib for 3 weeks had no effect on body weights in the PDX mouse model (Fig. S2). This result suggested that in clinical practice, the agent will be easily tolerated by patients with ICC.

Based on transcriptome analysis, we found that the cell cycle and VEGF signaling pathways were significantly enriched (Fig. 4). We speculated that inhibition of the VEGF/PI3K/AKT signaling pathway is the primary way that anlotinib causes tumor cell cycle arrestment, and inhibits proliferation, apoptosis, and invasion. Anlotinib strongly inhibits multiple targets, such as VEGFR, PDGFR, FGFR, and c-kit^11^. Based on the transcriptome analysis, we evaluated the protein levels and phosphorylation of VEGFR2, FGFR, and PDGFR-β. Only the VEGFR2 phosphorylation level was significantly reduced after anlotinib treatment. The results of further investigation supported the hypothesis that the therapeutic effect of anlotinib might mainly be via inhibition of the phosphorylation level of VEGFR2 and then affecting PIK3/AKT signal activation. This process then caused the changes in proliferation, migration, and invasion in the tumor cells (Fig. 6). Moreover, preventing AKT signaling activation is considered as a promising strategy for enhancing treatment efficiency of TKI including sorafenib and lenvatinib in solid cancer including HCC^33,34^. Therefore, we speculated that application Anlotinib might greatly benefit the treatment potentials of other TKIs including sorafenib or Lenvatinib. Importantly, more efforts should be made to clarify the efficiencies of this strategy, and to find the optimal combination to achieve the best treatment response. The results of using anlotinib in PDX models indicated that the excellent response (total average TGI 95.2%) to anlotinib in the six ICC PDX models was acquired. High levels of VEGFR2 expression were detected in tumor tissues of six PDX models; this result might explain the high response rate of anlotinib (Figs. 3b and S5B). The agreement between the results for VEGFR2 expression in tumor tissues before anlotinib administration and the TGI of anlotinib in six ICC PDX models suggested that VEGFR2 would be a useful indicator for predicting therapeutic response to anlotinib in patients with ICC (*P* = 0.046, *r* = 0.671; Fig. S5B). Thus, these results suggested that VEGFR2 might be a useful therapeutic target and indicator during use of anlotinib treatment in patients with ICC.

Based on the results that knockdown of VEGFR2 greatly attenuated treatment efficiency, we identified VEGFR2 as an important target of anlotinib. As a multi-target agent, anlotinib might exert its effects via other pathways or molecules. This possibility remains to be investigated. Our PDX model-based investigations provided attractive preclinical results. Therefore, a phase-I or -II prospective study should be performed to evaluate the efficacy of anlotinib treatment in patients with ICC. Before clinical use of anlotinib in patients with ICC, more data should be collected to verify its antitumor activities, side effects, and adverse events. Only then can anlotinib be used efficiency, safely, and extensively.

In conclusion, based on results from in vitro and PDX models, this study found that anlotinib had excellent antitumor activity in ICC cells. It also revealed that the antitumor effect of anlotinib was mainly via inhibition of VEGFR2 phosphorylation levels and the subsequent effects on activation of the PIK3/AKT signal to cause changes in proliferation, apoptosis, and invasion of tumor cells.

## Materials and methods

### Reagents

Anlotinib was provided by Zhengda Tianqing Pharmaceutical Group Co., Ltd. Dimethyl sulfoxide (Sigma, St Louis, MO, USA) was used to dissolve the drug. Matrigel was purchased from BD Bioscience (Pasadena, CA, USA). Working solutions were prepared by diluting the stock solution with RPMI 1640. The final DMSO concentration was 0.1%.

### ICC cell lines, transfection, and RNA-Seq analysis

The HCC9810 (Chinese Academy of Sciences Shanghai Branch Cell Bank, Shanghai, China) and RBE (Cell Resource Center of Tohoku University, Tohoku, Japan) human ICC cell lines were used in this study^16^. The cell lines were grown in RPMI 1640 with 10% fetal bovine serum (FBS) (Gibco) and antibiotics (penicillin 100 U/ml, streptomycin 100 mg/ml) in a 37 °C humidified atmosphere with 5% CO_2_. HCCC9810 and RBE cells were used in the siRNA analysis, and transfection of siRNA was performed using Lipofectamine 3000 (Invitrogen) according to the manufacturer’s instructions.

The target siRNA sequences were: siVEGFR2-1 5′-GCCACCAUGUUCUCUAAUATT-3′, siVEGFR2-2 5′-GCGGCUACCAGUCCGGAUATT-3′, and siNC 5′-UUCUCCGAACGUGUCACGUTT-3′. The RNA duplexes were synthesized by Genomeditech (Shanghai, China).

HCCC9810 and RBE cells were cultured with or without anlotinib for 24 h. The total RNA of the cultured cells was extracted using the RNeasy kit (Qiagen, Hilden, Germany) according to the manufacturer’s instructions. RNA was quantified using the Qubit^®^ RNA HS Assay Kit by Qubit^®^2.0 Fluorometer (Life Technologies, Grand Island, NY, USA). RNA quality was analyzed using an Agilent 2100 bioanalyzer (Agilent Technologies, Palo Alto, CA. USA). RNA-seq library construction for next-generation sequencing and paired-end deep sequencing was performed on an Illumina PE150 platform (Illumina, San Diego, CA), according to the manufacturer’s protocol.

### Cell proliferation, migration, and invasion assays

The Cell Counting Kit-8 (Dojindo, Kumamoto, Japan) was used to measure inhibitory effects on cell proliferation. The human ICC cell lines HCCC9810 and RBE were cultured in 96-well plates at a density of 5000 cells per well. They were incubated for 8 h and then treated with or without anlotinib at the indicated concentrations for 24, 48, or 72 h. The tumor cells were then washed twice with PBS and treated with CCK-8 reagent at a 1:10 dilution and incubated for 2 h at 37 °C. Absorbance at 450-nm was detected every 24 h to generate growth curves.

The wound-healing assay was performed as previously described^35^. Briefly, HCCC9810 and RBE cells were cultured with FBS-free tumor supernatant in 6-well plates and were incubated until they reached 90% confluence. A scratch was created across the center of the cell layer using a sterile 100-μl pipette tip. After 24 h, photographs were taken under the microscope, and cell migration distance was calculated using Image J software.

Matrigel invasion (with or without matrigel) was performed as previously described^35^. Briefly, 2 × 10^6^ HCCC9810 and RBE cells from each group were seeded in the upper chamber in FBS-free RPMI 1640. The chamber was coated with a matrix gel (for invasion assay, 1:8 diluted, Corning, ME) or without matrigel (for migration assay). RPMI 1640 containing 20% FBS was added to the lower chamber as a chemo-attractant. Mitomycin C was added to the upper chamber to stop cell proliferation. The tumor cells were then treated with or without anlotinib (5 µM). After 12 (for the migration assay) and 48 h (for the invasion assay) of incubation, tumor cells that had invaded to the lower surface of the membrane were fixed using 4% methanol, stained with crystal violet, and counted in five random ×100 microscopic fields per sample. All assays were performed in triplicate.

### Colony formation, cell cycle, and apoptosis assays

For the colony formation assay, HCCC9810 or RBE cells were placed into 6-well plates at a density of 1000 cells/well and incubated with different concentrations of anlotinib for 14 days. After staining with 0.1% crystal violet for 30 min, the colonies were visualized and quantified. For the cell cycle assays, tumor cells were treated with anlotinib at indicated concentrations for 24 h, and cell cycle distribution was analyzed using FCM with PI/RNase staining buffer (BD Biosciences). For the apoptosis assays, cells were incubated with anlotinib at indicated concentrations for 48 h^13^. The cells were harvested and then detected using FCM after staining with annexin V-FITC and propidium iodide. All assays were performed in triplicate.

### Quantitative real-time polymerase chain reaction (qRT-PCR) and western blot analyses

Total RNA was extracted from cultured cells using TRIzol reagent (Invitrogen, USA). It was reverse transcribed into cDNA using the PrimeScript RT kit (Takara, Japan) according to the manufacturer’s instructions^36^. The cycling conditions were 95 °C for 10 s, 60 °C for 20 s, and 72 °C for 20 s. Each reaction was repeated three times. The primer sequences for RT-PCR are shown in Table S1. Western blotting was performed as previously described^37,38^. The primary antibodies are listed in Table S2.

### PDX animal models

PDX models were constructed as described in our previous study^39^. Six ICC cases with successful construction were resuscitated and implanted under the skin of nonobese diabetic-severe combined immunodeficiency mice. When the subcutaneous tumor was 1 cm in diameter, it was minced into pieces (~3 × 3 × 3 mm) and then subcutaneously implanted into the flanks of 4- to 5-week-old nude mice. After the tumor volume reached 100–200 mm^3^, the mice were randomly divided into two groups: (1) control with PBS administration (100 μl, intragastric administration, qd) and (2) anlotinib (3 mg/kg, intragastric administration, qd). Mouse health status and tumor growth were observed daily. Tumor volume and mouse body weight were recorded twice per week. Eighteen days after drug intervention, the mice were sacrificed using dislocation, and the tumor tissues were harvested to measure tumor weights and volumes. Tumor size was measured using a caliper, and tumor volume was calculated using: tumor volume = 0.5 × length × (width)^2^ as we previously described^39^. The antitumor effect of anlotinib was evaluated using TGI. The TGI (%) calculation was: TGI (%) = [1 − (tumor volume at the end of the anlotinib treatment group period − tumor volume at the beginning of the anlotinib treatment group period)/(tumor volume at the end of the control group period − the tumor volume at the start of the control group period)]. All experiments were approved by the Research Ethical Committee of Zhongshan Hospital.

### Terminal deoxynucleotidyl transferase-mediated deoxyuridine triphosphate nick-end labeling (TUNEL), immunohistochemistry, and IF assays

Formalin-fixed PDX tumor tissue samples were embedded in paraffin and cut into 4-μm sections. Tumor sections were partially stained with H&E, and others were stained with PCNA (1:300), E-cadherin (1:300), and N-cadherin (1:800) for IHC analysis as previously described^40^. Images were captured using a microscope (Nikon Eclipse Ti-SR, Japan). Meanwhile, TUNEL assays (Roche, Indianapolis, IN) were performed to evaluate apoptosis rate after anlotinib treatment, following the manufacturer’s instructions.

IF was used to detect target protein position and expression, as previously described^41^. Briefly, after fixing with 4% paraformaldehyde and blocking with 5% FBS, samples were incubated with primary antibodies (E-cadherin 1:50, N-cadherin 1:100) at 4 °C overnight, followed by incubation with secondary antibody (Biyuntian, Shanghai, China). The nuclei were counterstained with DAPI. The intensity of fluorescence was detected using a fluorescence microscope (Nikon Eclipse C1, Japan).

### Statistical analysis

Statistical analyses were performed with IBM SPSS 19.0 software (SPSS, Chicago, IL) as previously described^42^ and with GraphPad Prism 7 for Windows (GraphPad Software Inc, San Diego, CA, USA). The results for continuous variables were presented as mean ± standard deviation values. Student’s *t* tests or Wilcoxon signed-rank tests were used for between-group comparisons. Categorical data were analyzed using chi-squared tests or Fisher’s exact tests. *P* values < 0.05 (two-tailed) were considered to indicate statistically significant results. All triplicate results were quantifications of independent experiments. The complete dataset is available as GEO proles on the GEO (Gene Expression Omnibus) database (https://www.ncbi.nlm.nih.gov/geo/query/acc.cgi?acc=GSE149901).

## Supplementary information

Supplementary Table 1

Supplementary Table 2

Supplementary Figure legends

Figure S1

Figure S2

Figure S3

Figure S4

Figure S5
