# Supplementary material for: Anlotinib suppresses tumor progression via blocking the VEGFR2/PI3K/AKT cascade in intrahepatic cholangiocarcinoma
Source: Cell Death Dis. 2020 Jul 24;11(7):573. doi: 10.1038/s41419-020-02749-7 (PMC7381674; doi:10.1038/s41419-020-02749-7)
Supplement: Supplementary file 3 — Supplementary Figure legends [file 41419_2020_2749_MOESM3_ESM.docx]

**Supplementary Figure S1.** (A) After 24 hours of Anlotinib treatment, the protein levels of EMT (E-cadherin, N-cadherin, Vimentin, αSMA, CK19) were detected by Western blotting (Left); Densitometry analysis was performed on three experiments representative and expressed relative to GAPDH or the corresponding total protein as the internal control (Right). (B) qRT-PCR (right) analyses were used to detect the expression of EMT markers (E-cadherin, N-cadherin, Vimentin, αSMA, CK19). All bar graphs depict quantification of triplicate results with mean ± SD values. ***, P < 0.001.

**Supplementary Figure S2.** (A) The images show the sizes of six PDX model tumors at the end of the experiment; (B) Mouse weights were calculated twice a week. Each point represents mean ± SD values for body weight in each group.

**Supplementary Figure S3.** Results for proliferation (PCNA) rate, apoptosis (TUNEL) index, and staining intensity of E-cadherin and N-cadherin in six PDX models. (**, P < 0.01; ***, P < 0.001).

**Supplementary** **Figure S4.** (A) and (B) Densitometry analysis was performed on three experiments representative of Figure 5A and B. Results were expressed relative to GAPDH or the corresponding total protein as the internal control. All bar graphs depict quantification of triplicate results with mean ± SD values. **, P < 0.01; ***, P < 0.001. (Image J 1.46r software; National Institutes of Health, Bethesda, MD).

**Supplementary Figure S5.** (A) Expression of FGFR1, p-FGFR1, PDGFR-β, and p-PDGFR-β in ICC cells after treatment with concentration gradient anlotinib for 24 hours was detected using immunoblotting. Similar results were obtained in three independent experiments. (Image J 1.46r software; National Institutes of Health, Bethesda, MD); (B) Expression of VEGFR2 in six PDX models were detected by western blotting; (C) Statistics for the correlation between VEGFR2 and their respective TGI% in six PDX tumors (P = 0.046, R^2^ = 0.671).

**Supplementary Figure S6.** (A) and (B) Densitometry analysis was performed on three experiments representative of Figure 7A and expressed relative to GAPDH or the corresponding total protein as the internal control. All bar graphs depict quantification of triplicate results with mean ± SD values. **, P < 0.01; ***, P < 0.001. (Image J 1.46r software; National Institutes of Health, Bethesda, MD).

.
