# Supplementary figures and images for: Anlotinib suppresses tumor progression via blocking the VEGFR2/PI3K/AKT cascade in intrahepatic cholangiocarcinoma
Source: Cell Death Dis. 2020 Jul 24;11(7):573. doi: 10.1038/s41419-020-02749-7 (PMC7381674; doi:10.1038/s41419-020-02749-7)

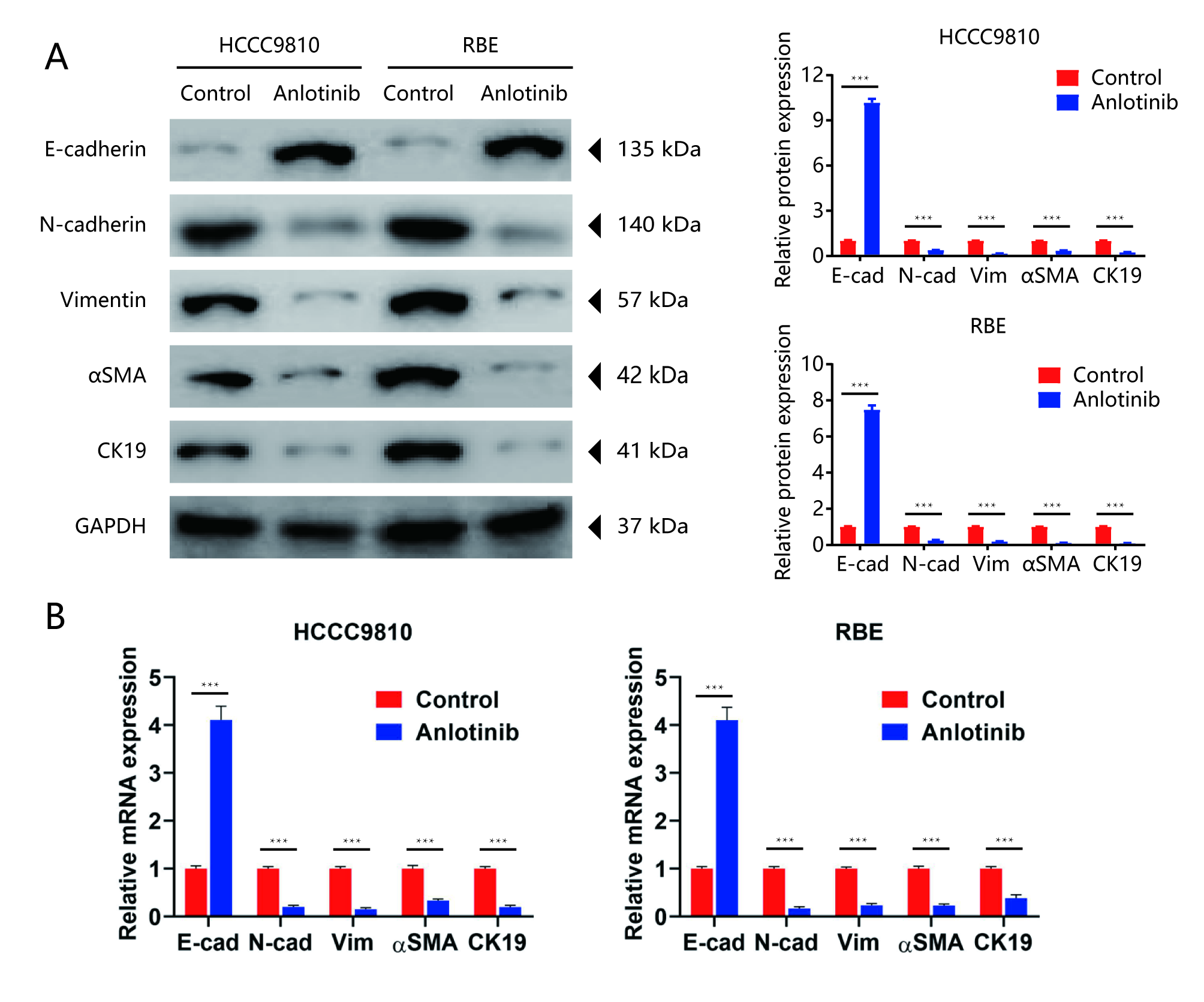

Supplement: Supplementary file 4 — Figure S1 [file 41419_2020_2749_MOESM4_ESM.tif]

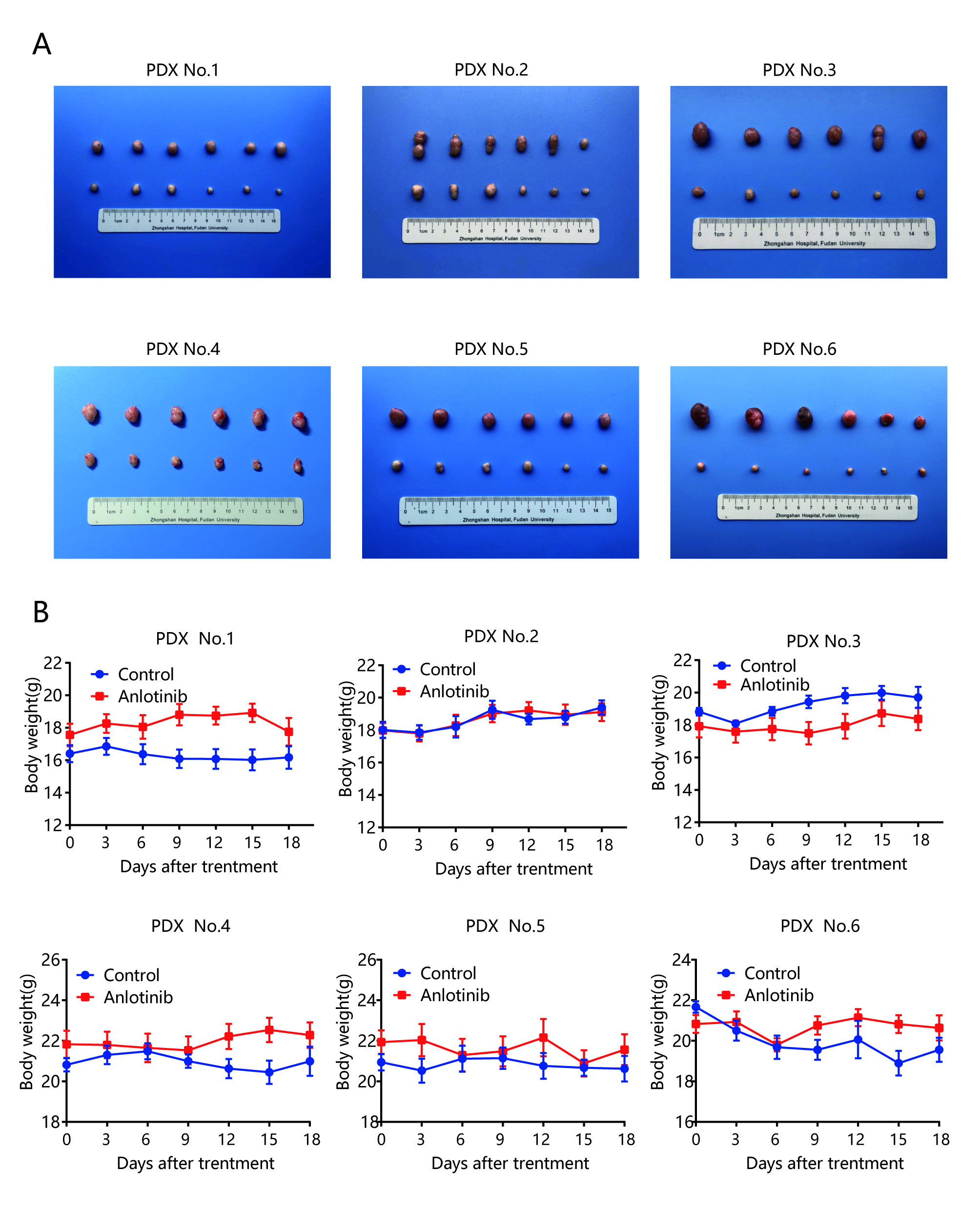

Supplement: Supplementary file 5 — Figure S2 [file 41419_2020_2749_MOESM5_ESM.tif]

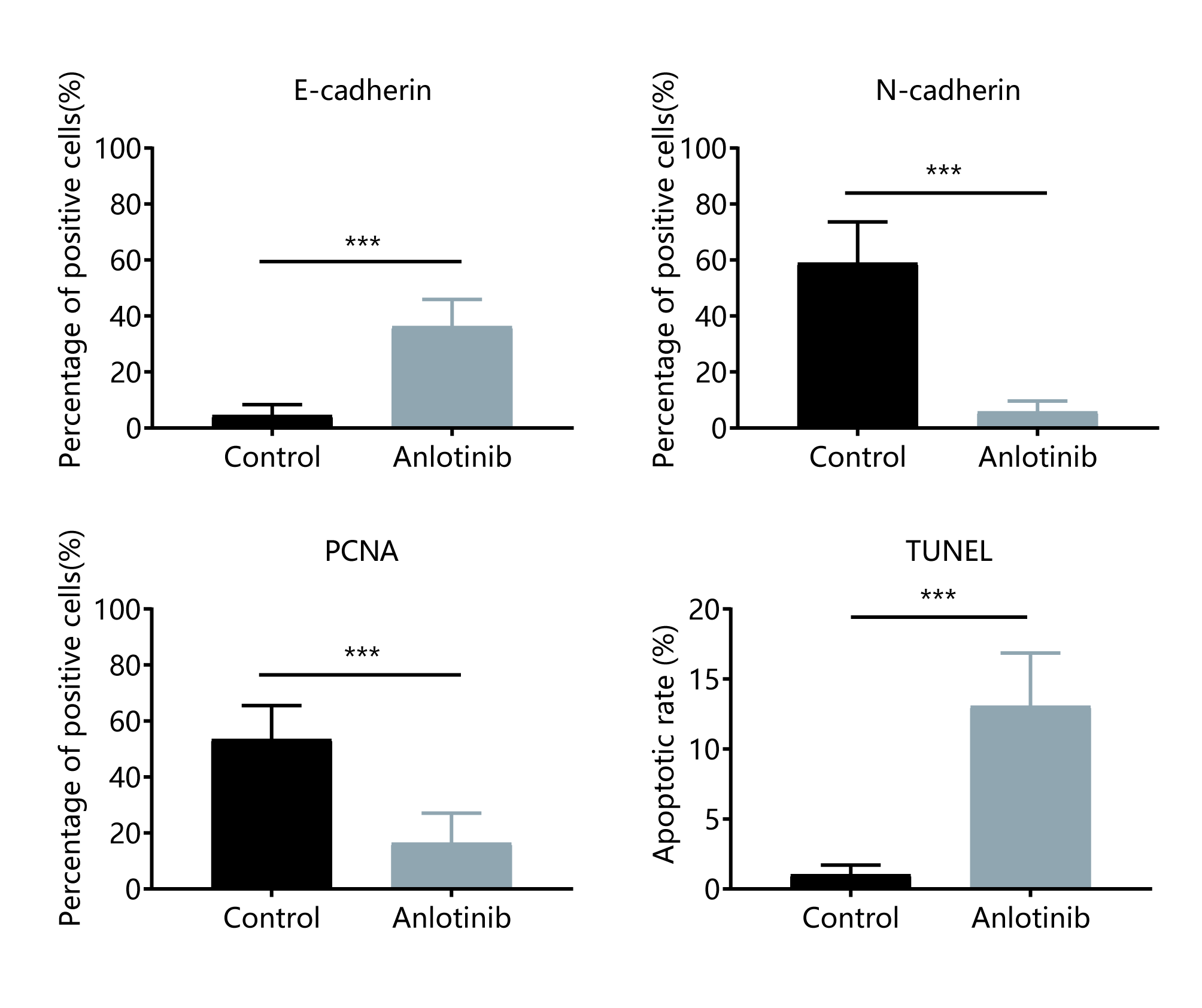

Supplement: Supplementary file 6 — Figure S3 [file 41419_2020_2749_MOESM6_ESM.tif]

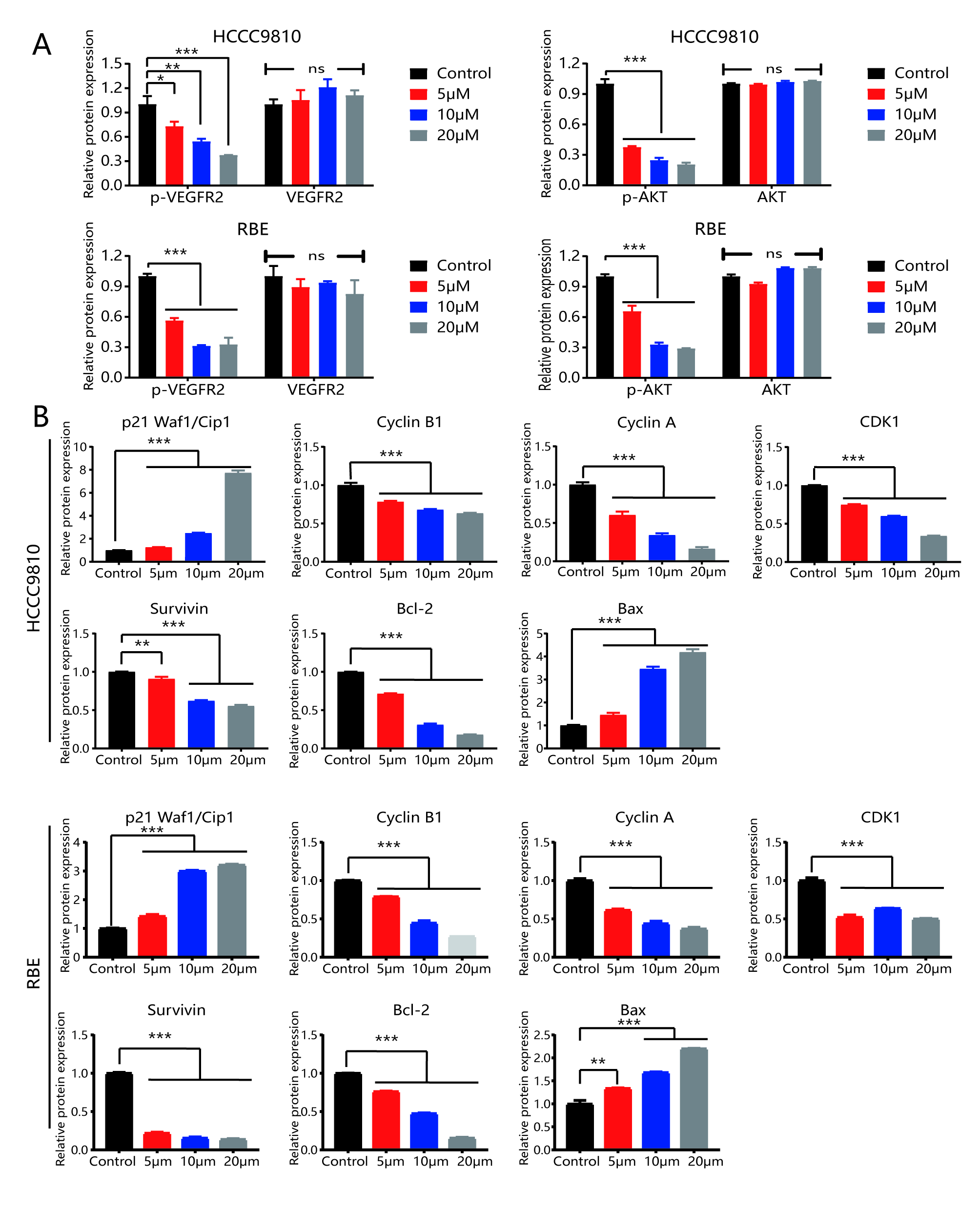

Supplement: Supplementary file 7 — Figure S4 [file 41419_2020_2749_MOESM7_ESM.tif]

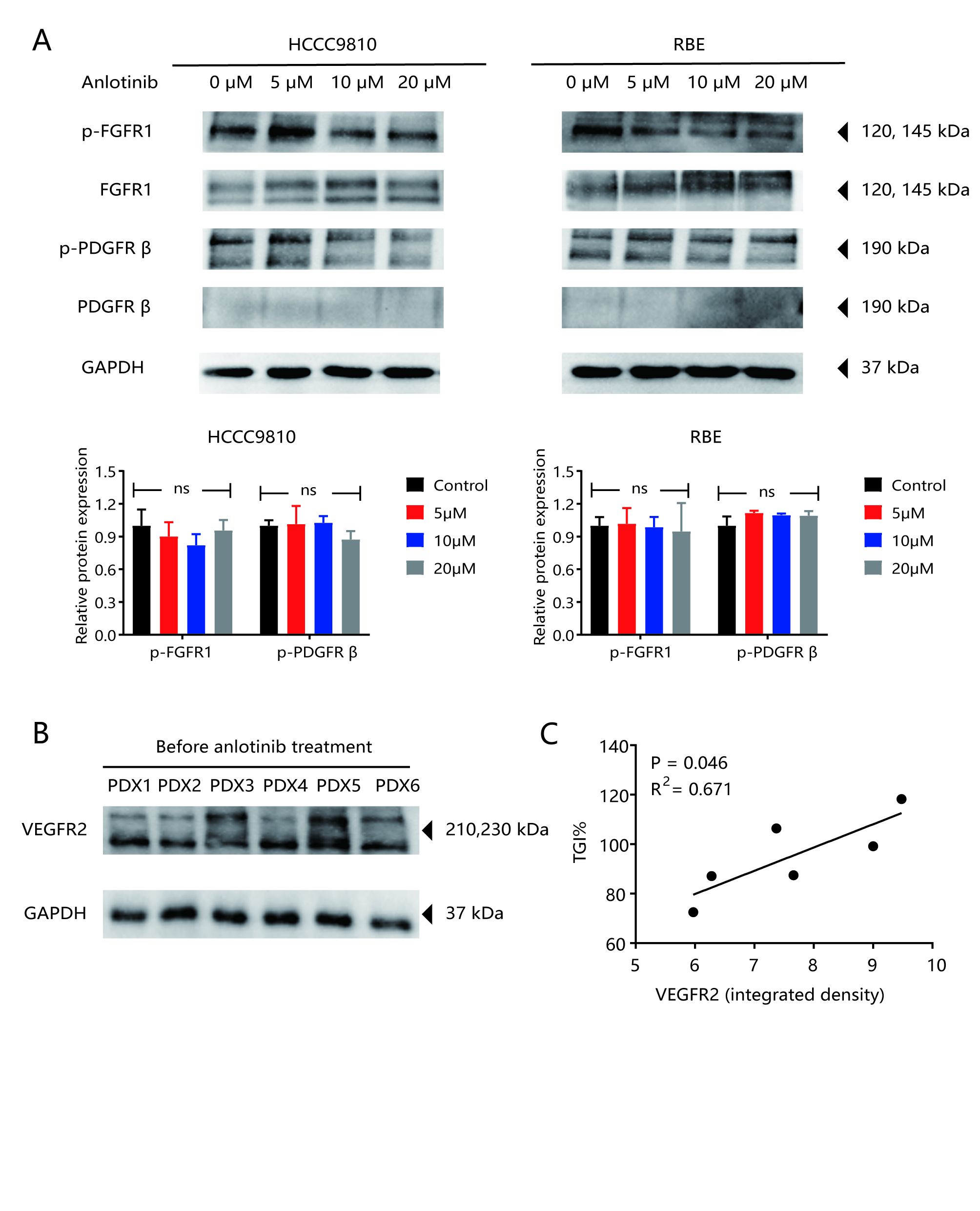

Supplement: Supplementary file 8 — Figure S5 [file 41419_2020_2749_MOESM8_ESM.tif]
